# Exploratory analysis of non-linear clozapine dose-concentration relationship in real-life hospital database

**DOI:** 10.3389/fphar.2025.1735337

**Published:** 2026-01-13

**Authors:** Anna Mach, Przemysław Bieńkowski, Szymon Tyras, Anna Wnorowska, Maria Radziwoń-Zaleska, Marcin Siwek, Marcin Wojnar

**Affiliations:** 1 Department of Psychiatry, Medical University of Warsaw, Warsaw, Poland; 2 Department of Biological and Community Psychiatry, Jagiellonian University Medical College, Cracow, Poland; 3 Department of Psychiatry, Addiction Center, University of Michigan, Ann Arbor, MI, United States

**Keywords:** clozapine, non-linear dose-concentration relationship, norclozapine, psychiatric inpatients, therapeutic drug monitoring

## Abstract

**Introduction:**

Clozapine (CLO) remains the gold standard for the treatment of drug-resistant schizophrenia. It is commonly accepted that there is a linear relationship between CLO dose and blood concentration, although deviations from this pattern are frequently observed in clinical practice. The aim of the present naturalistic study was to further investigate this relationship using a real-world database of CLO therapeutic drug monitoring (TDM) samples, with a particular focus on: i) identifying cases of “unexpected” CLO levels during repeated within-subject blood sampling in the process of CLO dose adjustment and ii) assessing linearity of the cross-sectional, between-subject CLO dose-concentration relationship and identifying potential breakpoints.

**Methods:**

The study was based on a single-center TDM database derived from routine monitoring of CLO concentration in psychiatric inpatients, supplemented with data from medical records. The database was reviewed independently by a laboratory medicine specialist and psychiatrist to identify individual cases of “unanticipated” dose-concentration relationships. The study also employed the Multivariate Adaptive Regression Splines (MARS) to detect non-linear relationships in the prediction of CLO levels, as determined by high-performance liquid chromatography. Analyses incorporated variables such as daily CLO dose, smoking status, age, sex, and co-medications.

**Results:**

Individual cases of “unanticipated” CLO concentrations supporting the partially non-linear within-subject dose-concentration relationship were unambiguously identified by both specialists. The MARS model revealed a breakdown in the between-subject CLO dose-concentration linear relationship identifying a hinge point around 400 mg/day, below which CLO concentrations were less dose-dependent. CLO dose and smoking were the most important predictive factors, but the model explained only about 25% of CLO concentration variability.

**Conclusion:**

Our data suggest that the non-linear relationship between CLO concentration and its daily dose can be a real-life clinical problem with CLO doses of ∼400 mg/day as the provisional hinge point. Although preliminary, the present results warrant further investigations on non-linear aspects of CLO pharmacology, including “unexpected” CLO concentrations, toxicity, and lack of therapeutic activity.

## 1 Introduction

Clozapine (CLO) is an atypical antipsychotic drug with proven efficacy in treatment-resistant schizophrenia (Kane et al., 1988; Siskind et al., 2016). Numerous studies have shown that clinical response to CLO is closely related to its blood levels, which better reflects the patient’s actual exposure to the drug than the daily dose (Wagner et al., 2021; Tralongo et al., 2023; Schoretsanitis et al., 2020). Despite clear recommendations for the use of therapeutic drug monitoring (TDM) to optimize CLO treatment (Tralongo et al., 2023; Schoretsanitis et al., 2020), in practice, therapeutic decisions are often based solely on dosage and clinical assessment (Schoretsanitis et al., 2020).

It is generally accepted that there is a linear relationship between the administered dose of CLO and its concentration in the blood of a given patient (Jiménez-Fernández et al., 2024; de Leon et al., 2020; Flanagan et al., 2023a). This assumption reflects the first-order kinetics that characterize most psychotropic agents, enabling predictable titration and the clinical use of concentration-to-dose ratios to guide individualized dosing. When linearity holds, stable metabolic conditions ensure proportional increases in drug exposure with dose escalation (Hiemke et al., 2018). However, our clinical observations indicate that the actual relationship between CLO dose and concentration is often much more complex. In everyday practice, we regularly observe cases of “unexpected” CLO concentrations that do not fit a simple linear model. In some patients, a significant increase in drug dose does not result in a proportional increase in concentration; on the contrary, a plateau or even a decrease in drug levels is observed in some individuals. In other cases, even small changes in dose lead to unpredictable concentration values, both too low and dangerously high. “Unexpected” or non-linear CLO dose-concentration relationship has been suggested by other authors (Flanagan et al., 2023a; Alfaro et al., 2001; Couchman et al., 2010; Chang et al., 1997). “Unexpectedly” low clozapine levels (200–250 ng/mL) were detected in two male, medication-compliant inpatients receiving CLO doses as high as 800–1,000 mg/day for at least 2 months (Alfaro et al., 2001). In contrast, “unanticipated”, high CLO concentrations were observed in 1.2% of samples from a TDM service with CLO levels >1,000 ng/mL at prescribed doses up to 150 mg/day, with no clues for overcompliance (Couchman et al., 2010). Such deviations from linearity carry important clinical implications. Subproportional increases in exposure may contribute to under-treatment, whereas supraproportional increases can produce disproportionate rises in blood levels and elevate the risk of concentration-dependent toxicity (de Leon et al., 2020; Couchman et al., 2010).

Based on literature data and our own observations, we assumed that the problem of difficult-to-interpret CLO concentrations exists in real-life clinical practice and cannot be explained by well-established variables associated with CLO concentrations. It is likely to occur more frequently at higher doses of the drug, but it cannot be ruled out that this phenomenon also occurs at lower doses (de Leon et al., 2020; Flanagan et al., 2023a; Hiemke et al., 2018).

The aim of the present study was two-fold. First, we reviewed our in-hospital database for representative cases of “unanticipated” changes in CLO concentrations during dose adjustment that did not conform to the assumption of within-subject linearity. Second, we conducted an exploratory analysis using the Multivariate Adaptive Regression Splines (MARS) (Friedman, 1991) to evaluate the assumption of linearity in the single-time-point, between-subject CLO dose-concentration association and to identify potential breakpoints [for similar biomedical applications of the MARS, see (Rockwood et al., 2017; Park and Kim, 2018; Lu et al., 2021; Yuan et al., 2025)]. This analysis was designed to reflect real-life conditions in which therapeutic decisions are made without access to prior TDM results for individual patients.

## 2 Materials and methods

### 2.1 Study design

The study procedures were reviewed and approved by the local ethics committee at the Medical University of Warsaw (approval no. AKBE/83/2021).

Records of inpatients chronically treated with clozapine with the aid of TDM at the Nowowiejski Psychiatric Hospital in Warsaw between 2016 and 2021, as described in our previous reports (Mach et al., 2024a; Mach et al., 2024b), were retrospectively reviewed. The TDM database contained multiple clozapine measurements for some patients collected across several hospitalizations. Only measurements obtained during inpatient stays were included in the analysis, which significantly reduces the risk of non-adherence to CLO and other prescribed medications, unrecognized exacerbation of somatic illness, and/or active dependence on psychoactive substances. Clinical diagnoses were established based on the World Health Organization’s International Statistical Classification of Diseases and Related Health Problems, 10th revision (ICD-10) (World Health Organization, 1992). Demographic and clinical data, including daily CLO dose, smoking status and use of concomitant medications, were obtained from medical records and TDM referrals completed by psychiatrists.

Using the same database, we conducted two independent analyses aligned with our study aims. First, to examine within-subject occurrences of “unexpected” CLO levels, we reviewed multiple time-point measurements from CLO-treated inpatients (methodology described in Section 2.2; results in Section 3.1). Second, for the cross-sectional, between-subject statistical analysis of the CLO dose–concentration relationship, we included only the first steady-state measurement from each patient in the full cohort (methodology in Section 2.3; results in Section 3.2).

### 2.2 Identification of representative cases of “unanticipated” CLO dose-concentration relationship (within-subject approach)

The database was independently reviewed by a board-certified laboratory medicine specialist and a psychiatrist to identify representative cases of “unanticipated” changes in CLO concentration following CLO dose modifications, in the absence of any well-established factors known to affect clozapine levels [e.g., laboratory error, recent introduction of a metabolic inducer or inhibitor, pneumonia, fever, liver or kidney disease (de Leon et al., 2020; Yuan et al., 2025; Mach et al., 2024a)]. Both researchers were blinded to patients’ personal data. As this study was exploratory in nature and current clinical guidelines (Mach et al., 2024b; World Health Organization, 1992) provide no clear recommendations for the identification or management of non-linear changes in clozapine concentrations, no predefined criteria for case selection were applied during the review process. However, only cases independently classified as “unanticipated” by both reviewers are presented. In each case, sequences of CLO and NCLO determinations across a range of CLO doses were analyzed to better characterize the nature of these “unanticipated” CLO concentration changes.

### 2.3 Analysis of CLO dose-concentration relationship using the MARS (between-subject analysis)

To verify the traditional assumption of linearity of CLO dose-concentration relationship we employed the MARS, a non-parametric regression technique (Friedman, 1991). MARS models non-linear relationships by fitting a series of piecewise linear regressions, with breakpoints (“knots”) indicating where the slope of the relationship changes.

Only participants (n = 156) with complete data for all selected variables (i.e., no missing values) were included in the final analysis with the MARS. Additionally, due to the naturalistic nature of the dataset and the requirement for independent observations in MARS modelling, we excluded repeated measurements from the same individuals, retaining only the first-ever, steady-state determination of CLO and norclozapine (NCLO) performed in our laboratory.

The analysis began with the calculation of descriptive statistics for the selected sample. We then assessed the associations between CLO dose and CLO/NCLO serum levels by computing both linear (Pearson) and monotonic (Spearman) correlation coefficients. Next, we developed two separate MARS models to predict CLO and NCLO serum levels (in ng/mL). Predictor variables, for each model, included CLO dose (in mg/day), age (in years), sex (male/female), smoking status (yes/no), concomitant use of β-blockers (yes/no), and concomitant use of any psychotropic medication (e.g., antipsychotic, mood stabilizer, antidepressant) other than CLO (yes/no). Predictor selection was guided by prior research on factors influencing clozapine metabolism (Flanagan et al., 2023a; Mach et al., 2024a; Flanagan et al., 2023b), as well as by the fact that these variables were readily available in clinical practice. Modelling was performed in R Core Team (2021) using the earth package (Milborrow, 2014; Milborrow et al., 2024). Models were fit using default settings, with Friedman’s mi value set to 1 (additive models). Model pruning was conducted via cross-validation (*nfold* = 5, *ncross* = 100), such that the final model retained the number of terms that maximized the mean out-of-sample *R* ^2^. For each model, we reported the proportion of variance explained (*R* ^2^) and the estimated regression coefficients for the selected terms. We also assessed the relative importance of predictors based on their contribution to the Generalized Cross-Validation (GCV) score in the final pruned model.

### 2.4 CLO and NCLO determination

Concentrations of CLO and its active metabolite, NCLO, were determined by high-performance liquid chromatography (HPLC) with UV detection (Shimadzu) as a part of standard CLO TDM in our clinical centre. A detailed description of the analytical method used has been published previously (Mach et al., 2024a; Mach et al., 2024b).

The data were obtained from patients who received daily doses of CLO individually adjusted to their clinical condition. Blood samples for CLO and NCLO concentration determination were collected only at pharmacokinetic steady state–after at least 7 days of unchanged drug dosing–and at the time of minimum concentration, i.e. 10–14 h after the last dose. Each determination was performed twice, in parallel with the laboratory’s internal quality control. The laboratory regularly participates in interlaboratory quality control programmes (LGC Standards) for the parameters determined. In the case of results deviating from the expected values, a detailed analysis was performed to exclude potential clinical, pre-laboratory, and laboratory errors. Factors such as incorrect sample labelling, incorrect collection, unexpected clinical events, technical failures, power outages, and incorrect internal control values were taken into account. The personnel responsible for sample collection were experienced, and sample transport within the centre was efficient and in accordance with the established protocol. None of the biological materials were stored. All determinations were performed on the day of collection.

## 3 Results

### 3.1 Identification of representative cases of “unanticipated” CLO dose-concentration relationship (within-subject approach)

Table 1 shows representative cases of patients with “unexpected” CLO dose-concentration relationship. The cases may illustrate a non-linear rise (case 2 and 5) or decrease (case 1 and 4) in CLO concentration as well as a flat dose-concentration curve (case 3).

**TABLE 1:** Representative cases of “unexpected” clozapine and norclozapine serum levels from TDM registry data of in-hospital laboratory.

Case^a^	Age	Sex	Smoking	Sample no.	Daily CLO dose [mg]	CLO levels [ng/mL]	NCLO levels [ng/mL]
1	39	M	No	1st	375	991	365
				2nd (after 8 days)	325	1,038	423
				3rd (after 20 days)	300	655	299
2	53	M	Yes	1st	600	348	194
				2nd (after 23 days)	700	833	336
3	48	F	No	1st	850	1,485	618
				2nd (after 27 days)	800	1,589	551
				3rd (after 7 days)	600	1,494	671
4	57	F	Yes	1st	700	1,161	432
				2nd (after 8 days)	550	650	280
				3rd (after 22 days)	575	510	235
5	55	M	Yes	1st	375	866	259
				2nd (after 7 days)	475	1,521	562

^a^ Cases represent psychiatric inpatients in the process of clozapine dose optimization. In each case, a single test result and/or a sequence of test results was identified as “unexpected” by a psychiatrist and a laboratory medicine specialist after excluding clinical factors (e.g., changes in smoking habits, severe infection, liver disease, new co-prescription of metabolic inhibitor/inducer, etc.) known to alter CLO, levels [for details, see Methods and (Hiemke et al., 2018; de Leon et al., 2025; de Leon et al., 2022; Siwek, 2015; Masood and Karim, 2020)]. CLO, clozapine; NCLO, norclozapine.

It should be noted that the representative cases of “unanticipated” changes in CLO concentrations during dose adjustment were intended to illustrate possible deviations from within-subject linearity, taking multiple measurements from a single patient into account. In contrast, the MARS models (see the next paragraph) were used to identify non-linear CLO dose–concentration relationships in a cross-sectional, between-subject setting, considering only the first measurement for each patient. Therefore, the two approaches should not be conflated.

### 3.2 Analysis of CLO dose-concentration relationship using the MARS (between-subject analysis)

#### 3.2.1 Descriptive statistics

Basic sociodemographic and clinical characteristics of the study group (n = 156) are presented in Table 2.

**TABLE 2:** Demographic and clinical characteristics of the study group (n = 156).

Variable	Value
Age (years)	Mean = 47.58, SD = 14.03, range: 18–75
Sex	Number of females = 81 (52%)
CLO dose (mg/d)	Mean = 363.04, SD = 147.12, range: 125–850
CLO serum level (ng/mL)	Mean = 500.60, SD = 338.22, range: 65–1753
NCLO serum level (ng/mL)	Mean = 218.92, SD = 177.25, range: 33–1,161
Smoking status (yes)	N = 88 (56%)
Concomitant use of β-blockers (yes)	N = 14 (9%)
Concomitant use of other psychiatric medications (yes)	N = 84 (54%)
Diagnosis (F20.0 according to ICD-10)	N = 141 (90%)

CLO, clozapine; NCLO, norclozapine.

#### 3.2.2 Correlations between CLO dose and CLO/NCLO serum concentrations

**FIGURE 1:**
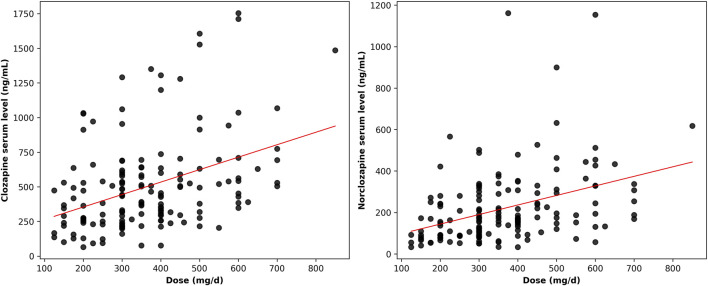
Scatterplots illustrating the relationship between clozapine dose and serum levels of clozapine and norclozapine.

Figure 1 presents scatterplots illustrating the associations between CLO dose and CLO/NCLO serum levels. CLO serum levels were moderately correlated with dose (Pearson’s r = 0.39, 95% CI: 0.25 to 0.52, p < 0.001; Spearman’s r = 0.38, 95% CI: 0.24 to 0.51, p < 0.001), as were NCLO levels (Pearson’s r = 0.38, 95% CI: 0.24 to 0.51, p < 0.001; Spearman’s r = 0.43, 95% CI: 0.29 to 0.55, p < 0.001).

#### 3.2.3 MARS modelling of CLO serum level

**FIGURE 2:**
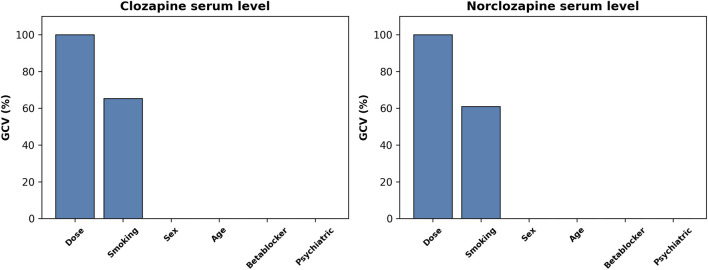
Bar plots of predictors importance in each of MARS models. MARS - Multivariate Adaptive Regression Splines; Dose - clozapine dose in mg/day; Betablocker - concomitant use of β-blockers; Psychiatric - concomitant use of any psychotropic medication other than clozapine; GCV, Generalized Cross-Validation score.

**FIGURE 3:**
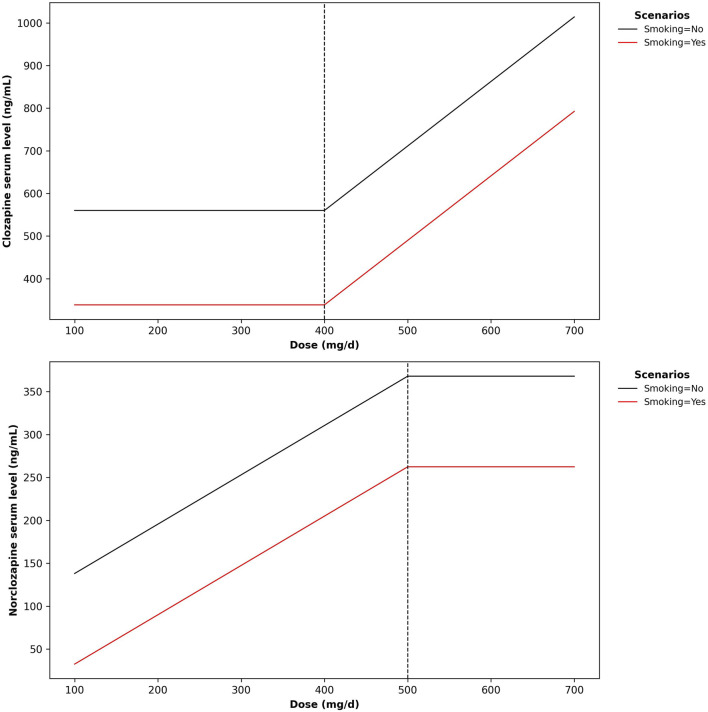
Modelled trajectories of clozapine and norclozapine serum levels across a clozapine dose range. Dose - clozapine dose in mg/day.

The MARS model predicting CLO serum level selected 3 out of 13 possible terms, involving 2 out of 6 predictors. The model achieved an *R* ^2^ of 0.25. The selected terms included the intercept (coefficient = 559.95817), smoking (coefficient = −221.49125), and a hinge function h (dose-400) (coefficient = 1.51357). Figure 2 (left subplot) displays the relative importance of predictors based on their contribution to the GCV score. A non-linear effect was identified for CLO dose as the predictor variable, with a hinge located at 400 mg/day (Figure 3, upper plot).

#### 3.2.4 MARS modelling of NCLO serum level

The MARS model predicting NCLO serum level selected 3 out of 13 possible terms, involving 2 out of 6 predictors. The model achieved an *R* ^2^ of 0.23. The selected terms included the intercept (coefficient = 367.92335), smoking (coefficient = −105.54943), and a hinge function h (500-dose) (coefficient = −0.57459). Figure 2 (right subplot) displays the relative importance of predictors based on their contribution to the GCV score. A non-linear effect was identified for CLO dose as the predictor variable, with a hinge located at 500 mg/day (Figure 3, bottom plot).

## 4 Discussion

In the present study, the representative cases of individual patients with difficult-to-interpret CLO levels were identified in order to validate previous case reports and to illustrate within-subject deviations from linearity (Alfaro et al., 2001; Couchman et al., 2010). The MARS models were also used for the identification of non-linear CLO dose-concentration relationships in a cross-sectional, between-subject setting mimicking real-life challenges of CLO dose adjustments without TDM data. To the best of our knowledge, this is the first study employing the MARS to analyse the CLO dose-concentration relationship and to identify non-linear associations in real-life clinical practice.

Moderate predictive performance was obtained for the MARS models with the *R* ^2^ value of 0.25 for CLO and 0.23 for NCLO. The analyses included variables with well-documented relevance for CLO metabolism, such as age, sex, CLO dose, smoking status, and the impact of concomitant medications other than CLO (β-blockers or other psychotropic drugs) (Flanagan et al., 2023c; Albitar et al., 2020; Singh et al., 2015; de Leon et al., 2025). As might be expected, the strongest predictors in both models were CLO dose and smoking status. Despite incorporating the well-established set of clinical variables (Flanagan et al., 2023c; Albitar et al., 2020; Singh et al., 2015; de Leon et al., 2025), a significant proportion of the variance in CLO concentrations remained unexplained. This finding indicates that even the well-established variables may not predict CLO concentrations with reasonable accuracy in some real-life TDM datasets.

The main finding of the MARS modeling was the identification of a non-linear relationship between CLO dose and serum concentration, along with the detection of a provisional hinge point. The hinge point was estimated at approximately 400 mg/day, suggesting that within the moderate-to-high dose range, increases in CLO dose are associated with a roughly linear rise in serum concentration (∼1.5 ng/mL per 1 mg of drug). Below this hinge point, the relationship weakens, and CLO levels become less predictable on the basis of CLO dose.

It is accepted that the relationship between the dose of CLO and its blood concentration is mostly linear, which forms the basis for estimating the individual optimal dose (de Leon et al., 2020). This means that in the absence of changes in factors affecting metabolism (i.e., without the presence of inducers or inhibitors), the concentration-to-dose ratio (C/D ratio) remains constant for a given patient (de Leon et al., 2022). However, the literature data are not consistent as to the extent to which such linearity indeed occurs (de Leon et al., 2025; Cheng et al., 1988; Choc et al., 1990; Schoretsanitis et al., 2021). In one of the early pharmacokinetic studies, Guitton et al. suggested that CLO exhibits linear kinetics in the concentration range of 145–1,411 ng/mL (Guitton et al., 1998). Other researchers have suggested that the relationship between CLO dose and concentration can be considered stable, provided that two criteria are met - no changes in factors that induce or inhibit metabolism and a CLO concentration above 150 ng/mL (Jiménez-Fernández et al., 2024). International guidelines also assume a linear relationship between CLO dose and concentration within the therapeutic range of CLO doses and in the absence of metabolism inducers or inhibitors (de Leon et al., 2022). It is worth noting that the range of CLO therapeutic doses may vary depending on ethnic origin or metabolic rate (de Leon et al., 2022; EMEA, 2002; Schoretsanitis et al., 2024). A recent experts’ opinion indicates that linear kinetics most often apply to the CLO concentration range of 150–1,000 ng/mL, if there is no influence of inducers or inhibitors (de Leon et al., 2025). Our results suggest the loss of linearity at lower doses with a critical value of approximately 400 mg/day. In clinical practice, the pharmacokinetics of the drug at lower doses may become less predictable and even a small modification of the dose may lead to a disproportionate change in drug concentration. Our finding may be related to the postulated non-linear increase in the risk of side effects (Couchman et al., 2010; Kikuchi et al., 2025), especially in the early phase of CLO dose adjustment. Previous studies have also shown non-linear pharmacokinetics at high CLO concentrations (>1,000 ng/mL) at which CLO metabolism is likely to approach saturation (de Leon et al., 2025). We did not observe a loss of linearity at high doses, which may be due to the smaller number of samples with high CLO concentrations (>1,000 ng/mL) in our database.

The loss of linear pharmacokinetics at lower CLO doses could be explained by several mutually non-exclusive factors, including complex metabolic pathways of the drug. Clozapine is metabolised in the liver, mainly by demethylation to NCLO and oxidation to N-oxide clozapine (Schoretsanitis et al., 2019). Cytochromes P450 isoenzymes, including CYP1A2, CYP3A4, CYP2C9, CYP2C19, and CYP2D6, play a key role in CLO demethylation (de Leon et al., 2022; Bertilsson et al., 1994; Fang et al., 1998). However, literature data are inconsistent regarding the percentage contribution of individual CYP enzymes to CLO metabolism (Thorn et al., 2018; Olesen and Linnet, 2001). The contribution of CYP isoforms varies depending on the concentration of the substrate. At low CLO concentrations, CYP1A2 is estimated to account for 70% of CLO metabolism, CYP2C19 and CYP3A4 for 20%–25%, with a small contribution from other CYP isoenzymes. However, at high drug concentrations, the contribution of cytochromes to CLO metabolism shifts in favour of CYP3A4 and CYP2C19 (Olesen and Linnet, 2001). The predominance of highly inducible CYP1A2 at lower CLO concentrations may underlie the greater variability and lack of linear association between CLO dose and concentration observed for lower doses. For example, it is possible that even minor day-to-day fluctuations in the number of cigarettes smoked and/or changes in the depth of inhalation of smoke could increase this variability by altering the degree of enzyme induction (Tralongo et al., 2023; Flanagan et al., 2023a).

The MARS model also revealed a non-linear dose-concentration relationship for NCLO, although with a different pattern. The hinge point was located at slightly higher doses (approximately 500 mg/day) beyond which the NCLO concentration became less dependent on the CLO dose, possibly reflecting saturation of the CYP1A2 pathways responsible for the formation of the metabolite. NCLO has some pharmacological activity (Costa-Dookhan et al., 2020; Park et al., 2020; Jessurun et al., 2022) and may contribute to the occurrence of adverse reactions associated with CLO (Schoretsanitis et al., 2019; De Leon et al., 2003). For this reason, the non-linear relationship between CLO dose and NCLO concentration may make monitoring of treatment safety and predicting the risk of CLO side effects more difficult.

The present study has several limitations concerning both the case selection and the between-subject analysis. With regard to the case selection (the within-subject approach), although we made every effort to eliminate the most evident sources of laboratory error and the most plausible clinical explanations for the difficult-to-interpret cases of a non-linear clozapine dose–concentration relationship (Table 1), the selected cases may still reflect unrecognized sources of variability. These include potential issues related to blood sample handling, undiagnosed somatic conditions, missed doses of CLO or other medications, day-to-day fluctuations in the number of cigarettes smoked, variations in the depth of smoke inhalation, and - last but not least - various combinations of these and other unidentified biological or organizational factors. Because this study relied on a retrospective dataset, we were unable to simply repeat the measurements, which would be the most effective strategy for verifying “unexpected” results and therefore represents an important clue for future prospective research.

Regarding the between-subject analysis, our methodological choice of using MARS, carries both strengths and drawbacks. While MARS effectively captures non-linear patterns with high interpretability, its reliance on piecewise linear approximations may oversimplify more complex pharmacokinetic relationships. Moreover, model’s predictive performance was only moderate, suggesting that important sources of variability were missing. These may include not incorporated variables such as genetic factors (e.g., CYP polymorphisms), body composition, active inflammation, pharmacokinetic interactions (e.g., with caffeine or other dietary components), and subtle hepatic or renal dysfunction (Flanagan et al., 2023a; Diaz et al., 2018; Ammar et al., 2021; Alli-Balogun et al., 2023; Pardiñas et al., 2019; Ghoneim and Mansour, 2020; Hägg et al., 2000). Variables with potentially higher predictive power - such as genetic data - are, however, rarely available at the point of care. The moderate performance may also reflect the fact that some variables were coded only in binary form, which reduces granularity. For instance, the substantial difference between smoking one versus twenty cigarettes per day could not be represented. Likewise, we did not incorporate specific drug types (beyond two broad categories) or individual doses due to substantial heterogeneity. Finally, the lack of external validation of the model on non-training data prevents a robust assessment of generalizability. The retrospective design and single center setting further restrict external validity. Although our in-hospital TDM database reflects some aspects of real-world clinical practice, validation in larger, multicenter cohorts remains necessary. Importantly, our goal was not to build the most accurate or broadly generalizable model but to explore whether non-linear patterns emerge in the CLO dose-concentration relationship when examined alongside known, clinically accessible moderators in our sample. Accordingly, the presented model and hinge-point localization should be interpreted as preliminary indications of potential non-linearity rather than clinically applicable predictive tools.

In conclusion, the identified cases and the MARS analysis suggest that the non-linear relationship between CLO daily dose and its concentration can be a real-life clinical problem with CLO doses of approximately 400 mg/day as the provisional hinge point. Although preliminary, the present results warrant further investigations on non-linear aspects of CLO pharmacology, including “unexpected” CLO concentrations, toxicity, and lack of therapeutic activity.

## Data Availability

The raw data supporting the conclusions of this article will be made available by the authors, without undue reservation.
